# A Nuisance-Free Inference Procedure Accounting for the Unknown Missingness with Application to Electronic Health Records

**DOI:** 10.3390/e22101154

**Published:** 2020-10-14

**Authors:** Jiwei Zhao, Chi Chen

**Affiliations:** 1 Department of Biostatistics and Medical Informatics, University of Wisconsin-Madison, Madison, WI 53726, USA; 2 Novartis Institutes for Biomedical Research, Shanghai 201203, China; chi-2.chen@novartis.com

**Keywords:** nuisance, post-selection inference, missingness mechanism, regularization, asymptotic theory, unconventional likelihood

## Abstract

We study how to conduct statistical inference in a regression model where the outcome variable is prone to missing values and the missingness mechanism is unknown. The model we consider might be a traditional setting or a modern high-dimensional setting where the sparsity assumption is usually imposed and the regularization technique is popularly used. Motivated by the fact that the missingness mechanism, albeit usually treated as a nuisance, is difficult to specify correctly, we adopt the conditional likelihood approach so that the nuisance can be completely ignored throughout our procedure. We establish the asymptotic theory of the proposed estimator and develop an easy-to-implement algorithm via some data manipulation strategy. In particular, under the high-dimensional setting where regularization is needed, we propose a data perturbation method for the post-selection inference. The proposed methodology is especially appealing when the true missingness mechanism tends to be missing not at random, e.g., patient reported outcomes or real world data such as electronic health records. The performance of the proposed method is evaluated by comprehensive simulation experiments as well as a study of the albumin level in the MIMIC-III database.

## 1. Introduction

A major step towards scientific discovery is to identify useful associations from various features and to quantify their uncertainties. This usually warrants building a regression model for an outcome variable and estimating the coefficient associated with each feature as well as the precision of the estimator. Besides the traditional regression with a small dimensionality, with advances in biotechnology, the modern high-dimensional regression usually posits a sparse parameter in the model, and then applies regularization to select the significant features in order to recover the sparsity. In particular, the post-selection inference could be challenging in a regularized regression framework. In this paper, our main interest is to consider a regression model where the outcome variable is prone to missing values. We study both the traditional setting where regularization is not needed and the modern one with regularization.

The missing data issue is an inevitable concern for statistical analysis in various disciplines ranging from biomedical studies to social sciences. In many applications, the occurrence of missing data is usually not the investigator’s primary interest but complicates the statistical analysis. The validity of any method devised for missing data heavily depends on the assumption of the missingness mechanism [1]. Unfortunately, those assumptions are largely unknown and difficult, if not infeasible, to be empirically tested. Therefore, one prefers to concentrate on analyzing the regression model for the outcome variable, while treating the mechanism model as a nuisance. A flexible assumption imposed at the minimum level on the mechanism would provide protection against model misspecification at this level.

While it is indeed promising to regard the missingness mechanism as a nuisance with a flexible assumption, a potential issue is the model identifiability problem if the mechanism contains missing-not-at-random cases, i.e., allowing the mechanism to depend on the missing values themselves. In the past few years, researchers have made great progress on this topic by introducing a so-called instrument. This instrument could be a shadow variable [2,3,4,5,6,7] or an instrumental variable [8,9]. Both approaches are reasonable and are suitable for different applications. In this paper, we adopt the shadow variable approach as it facilitates the interpretability of the regression model for the outcome. The details of the shadow variable approach will be articulated later throughout the paper.

Therefore, we proceed with a semiparametric framework where our primary interest is a parametric regression, e.g., a linear model, where the statistical task is to estimate the parameter of interest and conduct statistical inference (particularly post-selection inference for the setting with regularization). For the nuisance missingness mechanism, we only impose a nonparametric assumption without specifying a concrete form. We encode the shadow variable as *Z*, which is one component of the covariate X. In general, a shadow variable with a smaller dimensionality allows more flexibility of the missingness mechanism. Therefore, although it could be multidimensional, we only consider univariate *Z* throughout the paper. With all of these ingredients, we analyze a conditional likelihood approach which will eventually result in a nuisance-free procedure for parameter estimation and statistical inference.

There are at least two extra highlights of our proposed method that are worth mentioning. The first pertains to the algorithm and computation. Although it looks complicated at first sight, we show that, via some data manipulation strategy, the conditional likelihood function can be analytically written as the likelihood of a conventional logistic regression with some prespecified format. Therefore, our objective function can be readily optimized by many existing software packages. This greatly alleviates the computational burden of our procedure. Second, while the variance estimation under the traditional setting is straightforward following the asymptotic approximation, it is challenging for the setting with regularization. To resolve this problem, we present an easy-to-implement data-driven method to estimate the variance of the regularized estimator via a data perturbation technique. It is noted that the current literature on the inference procedure for regularized estimation in the presence of missing values is very scarce. The authors of [10,11,12] all considered the model selection problem under high dimensionality with missing data; however, none of them studied the post-selection inference in this context.

The remainder of the paper is structured as follows. In Section 2, we first layout our model formulation and introduce the shadow variable and the conditional likelihood. Section 3 details the traditional setting without regularization. We present our algorithm of how to maximize the conditional likelihood function, the theory of how to derive the asymptotic representation of our proposed estimator and how to estimate its variance. In Section 4, we devote ourselves to the modern setting where the sparsity assumption is imposed and the regularization technique is adopted. Both algorithm and theory as well as the variance estimation through the data perturbation technique are presented. In Section 5, we conduct comprehensive simulation studies to examine the finite sample performance of our proposed estimator as well as the comparison to some existing methods. Section 6 is the application of our method to the regression model for the albumin level which suffers from a large amount of missing values in the MIMIC-III study [13]. The paper is concluded with a discussion in Section 7.

## 2. Methodology

Denote the outcome variable as *Y* and covariate X. We assume $X={\left(U^{{}^{T}},Z\right)}^{{}^{T}}$ where U is *p*-dimensional and *Z* univariate, with detailed interpretation later. We consider the linear model

(1) $$\begin{array}{c} Y=\alpha +\beta^{{}^{T}}U+\gamma Z+\epsilon , \end{array}$$

where β is also *p*-dimensional, α and γ are scalars and the true value of γ, $\gamma_{0}$, is nonzero, $\epsilon ∼N(0,\sigma^{2})$. We consider the situation that *Y* has missing values while X is fully observed. We introduce a binary variable *R* to indicate missingness: R=1 if *Y* is observed and R=0 if missing. To allow the greatest flexibility of the missingness mechanism model, we assume

(2) $$\begin{array}{c} \mathrm{pr}(R=1|Y,X)=\mathrm{pr}(R=1|Y,U)=s(Y,U), \end{array}$$

where s(·) merely represents an unknown and unspecified function not depending on *Z*. We reiterate that, as the assumption (2), in a nonparametric flavor, does not specify a concrete form of s(·), one does not need to be worrisome of the mechanism model misspecification. Moreover, as it allows the dependence on *Y*, besides missing-completely-at-random (MCAR) and many scenarios of missing-at-random (MAR), the assumption (2) also contains various situations of missing-not-at-random (MNAR).

We term *Z* the shadow variable following the works in [5,6,7,14]. Its existence depends on whether it is sensible that *Z* and *R* are conditionally independent (given *Y* and U) and that *Y* heavily relies on *Z* (as $\gamma_{0}\neq 0$). There are many examples in the literature documenting that the existence of *Z* is practically reasonable. In application, a surrogate or a proxy of the outcome variable *Y*, which would not synchronically affect the missingness mechanism, could be a good choice for the shadow variable *Z*.

We assume independent and identically distributed observations $\left\{r_{i},y_{i},u_{i},z_{i}\right\}$ for i=1,…,N and the first *n* subjects are free of missing data. Now we present a s(·)-free procedure via the use of the conditional likelihood. Denote $V={\left(Y,U^{{}^{T}}\right)}^{{}^{T}}$. We start with

$$\begin{array}{c} \prod_{i=1}^{n}p\left(v_{i}|z_{i},r_{i}=1\right)=\prod_{i=1}^{n}\frac{s(v_{i})}{g(z_{i})}p\left(v_{i}|z_{i}\right), \end{array}$$

where $g\left(z_{i}\right)=\mathrm{pr}\left(r_{i}=1|z_{i}\right)=\int \mathrm{pr}\left(r_{i}=1|v\right)p\left(v|z_{i}\right)dv$ and p(·|·) is a generic notation for conditional probability density/mass function. If V were univariate, we denote A as the rank statistic of $\left\{v_{1},\ldots ,v_{n}\right\}$, then

(3) $$\begin{array}{ccc} \;\;\; & & \;\;\;\prod_{i=1}^{n}p\left(v_{i}|z_{i},r_{i}=1\right)=p\left(v_{1},\ldots ,v_{n}|z_{1},\ldots ,z_{n},r_{1}=\cdots =r_{n}=1\right)\\ \;\;\; & = & \;\;\;p\left(A|v_{\left(1\right)},\ldots ,v_{\left(n\right)},z_{1},\ldots ,z_{n},r_{1}=\cdots =r_{n}=1\right)p\left(v_{\left(1\right)},\ldots ,v_{\left(n\right)}|z_{1},\ldots ,z_{n},r_{1}=\cdots =r_{n}=1\right). \end{array}$$

The conditional likelihood that we use, the first term on the right hand side of (3), is exactly

(4) $$\begin{array}{ccc} & & p\left(A|v_{\left(1\right)},\ldots ,v_{\left(n\right)},z_{1},\ldots ,z_{n},r_{1}=\cdots =r_{n}=1\right)=\frac{p(v_{1},\ldots ,v_{n}|z_{1},\ldots ,z_{n},r_{1}=\cdots =r_{n}=1)}{p(v_{\left(1\right)},\ldots ,v_{\left(n\right)}|z_{1},\ldots ,z_{n},r_{1}=\cdots =r_{n}=1)}\\ & = & \frac{\prod_{i=1}^{n}p\left(v_{i}|z_{i},r_{i}=1\right)}{\Sigma_{\omega \in \Omega }\prod_{i=1}^{n}p\left(v_{\omega (i)}|z_{i},r_{i}=1\right)}=\frac{\prod_{i=1}^{n}p\left(v_{i}|z_{i}\right)}{\Sigma_{\omega \in \Omega }\prod_{i=1}^{n}p\left(v_{\omega (i)}|z_{i}\right)}, \end{array}$$

where Ω represents the collection of all one-to-one mappings from {1,…,n} to {1,…,n}. Now (4) is nuisance-free and can be used to estimate the unknown parameters in $p(v_{i}|z_{i})$.

Although V is multidimensional in our case, the idea presented above can still be applied and it leads to

(5) $$\begin{array}{c} \frac{\prod_{i=1}^{n}p\left(y_{i},u_{i}|z_{i},r_{i}=1\right)}{\Sigma_{\omega \in \Omega }\prod_{i=1}^{n}p\left(y_{\omega (i)},u_{\omega (i)}|z_{i},r_{i}=1\right)}=\frac{\prod_{i=1}^{n}p\left(y_{i},u_{i}|z_{i}\right)}{\Sigma_{\omega \in \Omega }\prod_{i=1}^{n}p\left(y_{\omega (i)},u_{\omega (i)}|z_{i}\right)}. \end{array}$$

Furthermore, to simplify the computation, we adopt the pairwise fashion of (5) following the previous discussion on pairwise pseudo-likelihood in [15], which results

$$\begin{array}{c} \prod_{1\leq i<j\leq n}\frac{p\left(y_{i},u_{i}|z_{i}\right)p\left(y_{j},u_{j}|z_{j}\right)}{p\left(y_{i},u_{i}|z_{i}\right)p\left(y_{j},u_{j}|z_{j}\right)+p\left(y_{i},u_{i}|z_{j}\right)p\left(y_{j},u_{j}|z_{i}\right)}. \end{array}$$

After plugging in model (1) and some algebra, the objective eventually becomes to minimize

(6) L(θ)=N2−1∑1≤i<j≤Nϕij(θ)=N2−1∑1≤i<j≤Nrirjlog{1+Wijexp(θTdij)},

where $\theta ={\left(\tilde{\gamma },{\tilde{\beta }}^{{}^{T}}\right)}^{{}^{T}}$, $\tilde{\gamma }=\gamma /\sigma^{2}$, $\tilde{\beta }=\tilde{\gamma }\beta$, $d_{ij}={\left(-y_{i\setminus j}z_{i\setminus j},u_{i\setminus j}^{{}^{T}}z_{i\setminus j}\right)}^{{}^{T}}$, $y_{i\setminus j}=y_{i}-y_{j}$, $u_{i\setminus j}=u_{i}-u_{j}$, $z_{i\setminus j}=z_{i}-z_{j}$ and $W_{ij}=p\left(z_{i}|u_{j}\right)p\left(z_{j}|u_{i}\right)/\left\{p\left(z_{i}|u_{i}\right)p\left(z_{j}|u_{j}\right)\right\}$.

Denote the minimizer of (6) as $\hat{\theta }$. By checking that

$$\frac{\partial^{2}\phi_{ij}\left(\theta \right)}{\partial \theta \partial \theta^{{}^{T}}}=r_{i}r_{j}{\left\{1+W_{ij}\exp\left(\theta^{{}^{T}}d_{ij}\right)\right\}}^{-2}W_{ij}\exp\left(\theta^{{}^{T}}d_{ij}\right)d_{ij}d_{ij}^{{}^{T}}$$

is positive definite, $\hat{\theta }$ uniquely exists. To compute $\hat{\theta }$, one also needs a model for $W_{ij}$. Fortunately, this model only depends on fully observed data $x_{i}$ and $x_{j}$. Essentially any existing parametric, semiparametric, or nonparametric modeling technique for p(z|u) can be used, and $W_{ij}$ can be estimated accordingly. Throughout, we denote ${\hat{W}}_{ij}$ as an available well-behaved estimator of $W_{ij}$. Although our procedure stems from p(y,u|z,r=1), which only relies on the data $\left\{y_{i},x_{i}\right\}$ with i=1, it can be seen that, not only the data $\left\{y_{i},x_{i}\right\}$ with i=1 are used to compute $\hat{\theta }$, the data $\left\{x_{i}\right\}$ with i=0 are also used in the process of estimating $W_{ij}$. Therefore, all observed data, both from completely-observed subjects and from partially-observed subjects, are utilized in our procedure.

One can notice that, due to the assumption (2) which allows the greatest flexibility of the mechanism model and the adoption of the conditional likelihood, not all parameters α, β, γ, and $\sigma^{2}$ are estimable. Nevertheless, the parameter β, which quantifies the association between *Y* and U after adjusting for *Z* and is of primarily scientific interest, can be fully estimable. The remainder of the paper focuses on the estimation and inference of β, as well as the variable selection procedure based on β.

Before moving on, we give some comparison with the existing literature to underline the novel contributions we make in this paper. Based on a slightly different but more restrictive missingness mechanism assumption that pr(R=1|Y,X)=a(Y)b(X), Refs. [16,17,18] used the similar idea to analyze non-ignorable missing data for a generalized linear model and a semiparametric proportional likelihood ratio model, respectively. They focused on different aspects of how to use the conditional likelihoods and their consequences such as the partial identifiability issue and the large bias issue. In this paper, we focus on the linear model (1) and we just showed that the parameter β is fully identifiable. It can be seen that the method presented in this paper can be applied to different models, but their identifiability problems or some other relevant issues have to be analyzed on a case-by-case basis. For instance, Ref. [19] studied the parameter estimation problem in a logistic regression model with a low dimensionality under assumption (2). They showed that, different from the current paper, all the unknown parameters are identifiable in their context. However, because of the complexity of their objective function, the algorithm studied in [19] is trivial and cannot be extended to a high dimensional setting.

## 3. Traditional Setting without Regularization

**Computation**. Directly minimizing L(θ) is feasible; however, it is very computationally involved. From rearranging the terms in L(θ), we realize that it can be rewritten as the negative log-likelihood function of a standard logistic regression model. To be more specific, let *k* be the index of pair (i,j) with k=1,…,K and K=n2. Then,

(7) $$\begin{array}{c} L\left(\theta \right)=\frac{1}{K}\sum_{k=1}^{K}\log\left\{1+\exp\left(s_{k}\theta^{{}^{T}}t_{k}+\log{\hat{W}}_{k}\right)\right\}, \end{array}$$

where $s_{k}=-\mathrm{sign}\left(z_{i\setminus j}\right)$, $t_{k}=\left(\right|z_{i\setminus j}|y_{i\setminus j},-|z_{i\setminus j}{\left|u_{i\setminus j}^{{}^{T}}\right)}^{{}^{T}}$. Denote $g_{k}=I\left\{z_{i\setminus j}>0\right\}$, then one can show that the summand in (7), $\log\left\{1+\exp\left(s_{k}\theta^{{}^{T}}t_{k}+\log{\hat{W}}_{k}\right)\right\}$, equals,

$$-\left[g_{k}\left(\theta^{{}^{T}}t_{k}+s_{k}\log{\hat{W}}_{k}\right)-\log\left\{1+\exp\left(\theta^{{}^{T}}t_{k}+s_{k}\log{\hat{W}}_{k}\right)\right\}\right],$$

which is the contribution of the *k*-th subject to the negative log-likelihood of a logistic regression with $g_{k}$ as the response, θ as the coefficient, $t_{k}$ as the covariate, and $s_{k}\log{\hat{W}}_{k}$ as the offset term, but without an intercept. Therefore, $\hat{\theta }$ can be obtained by fitting the aforementioned logistic regression model. Algorithm 1 describes the steps for data manipulation and model fitting to estimate θ under this traditional setting.
**Algorithm 1** Minimization of (6) without penalization1: **Inputs:**  $\left\{y_{i},u_{i},z_{i}\right\},\left\{y_{j},u_{j},z_{j}\right\},{\hat{W}}_{ij}$, for i=1,…,n and j=1,…,n 2: **Initialize:**  k←0 3: **for**
j∈{2:n}
**do** 4:  **for**
i∈{1:(j−1)}
**do** 5:    k←k+1 6:    $y_{i\setminus j}\leftarrow y_{i}-y_{j}$, $u_{i\setminus j}\leftarrow u_{i}-u_{j}$, $z_{i\setminus j}\leftarrow z_{i}-z_{j}$, ${\hat{W}}_{k}\leftarrow {\hat{W}}_{ij}$ 7:    $g_{k}\leftarrow I\left\{z_{i\setminus j}>0\right\}$ 8:    $s_{k}\leftarrow -\mathrm{sign}\left(z_{i\setminus j}\right)$ 9:    $t_{k}\leftarrow \left(\right|z_{i\setminus j}|y_{i\setminus j},-|z_{i\setminus j}{\left|u_{i\setminus j}^{{}^{T}}\right)}^{{}^{T}}$ 10: Fit logistic regression with response g, covariate t, offset $s^{{}^{T}}\log\hat{W}$, and no intercept. 11: **Outputs:**  $\hat{\theta }$


**Asymptotic Theory**. The asymptotic theory of $\hat{\theta }$ involves a model of p(z|u), which does not contain any missing values, and therefore any statistical model, either parametric, or semiparametric, or nonparametric, can be used. For simplicity, we only discuss the parametric case here, and any further elaborations will be rendered into Section 7. For a parametric model p(z|u;η), one can apply the standard maximum likelihood estimate $\hat{\eta }$. Here, we simply assume

(8) $$\begin{array}{c} \sqrt{N}\left(\hat{\eta }-\eta_{0}\right)=-G^{-1}\sqrt{N}\frac{1}{N}\sum_{i=1}^{N}\frac{\partial }{\partial \eta }\log\left\{p(z_{i}|u_{i};\eta_{0})\right\}+o_{p}\left(1\right), \end{array}$$

where $G=E\left[\frac{\partial^{2}}{\partial \eta \partial \eta^{{}^{T}}}\log\left\{p(z|u;\eta_{0})\right\}\right]$, $E∥\frac{\partial^{2}}{\partial \eta \partial \eta^{{}^{T}}}\log\left\{p(z|u;\eta_{0})\right\}{∥}^{2}<\infty$, $\eta_{0}$ is the true value of η, and $∥M∥=\sqrt{\mathrm{trace}(MM^{T})}$ for a matrix M. With this prerequisite, we have the following result for $\hat{\theta }$, and its proof is provided in Appendix A.

**Theorem** **1.** *Assume (8) as well as $E{∥\frac{\partial^{2}\phi_{ij}\left(\theta_{0},\eta_{0}\right)}{\partial \theta \partial \theta^{{}^{T}}}∥}^{2}<\infty$. Denote $\theta_{0}$ the true value of θ. Then*

$$\begin{array}{c} \sqrt{N}\left(\hat{\theta }-\theta_{0}\right)\overset{d}{\to }N\left(0,A^{-1}\Sigma A^{-1}\right), \end{array}$$

*where $A=E\left\{\frac{\partial^{2}\phi_{ij}\left(\theta_{0},\eta_{0}\right)}{\partial \theta \partial \theta^{{}^{T}}}\right\}$, $\Sigma =4E\left\{\lambda_{12}\left(\theta_{0},\eta_{0}\right)\lambda_{13}{\left(\theta_{0},\eta_{0}\right)}^{{}^{T}}\right\}$, $\lambda_{ij}\left(\theta_{0},\eta_{0}\right)=BG^{-1}M_{ij}\left(\eta_{0}\right)-N_{ij}\left(\theta_{0},\eta_{0}\right)$, $B=E\left\{\frac{\partial^{2}\phi_{ij}\left(\theta_{0},\eta_{0}\right)}{\partial \theta \partial \eta^{{}^{T}}}\right\}$, $M_{ij}\left(\eta_{0}\right)=\frac{1}{2}\left\{\frac{\partial }{\partial \eta }\log p\left(z_{i}|u_{i};\eta_{0}\right)+\frac{\partial }{\partial \eta }\log p\left(z_{j}|u_{j};\eta_{0}\right)\right\}$, and $N_{ij}\left(\theta_{0},\eta_{0}\right)=\frac{\partial \phi_{ij}\left(\theta_{0},\eta_{0}\right)}{\partial \theta }$.*

If one prefers the asymptotic result of $\hat{\beta }$, we have

**Corollary** **1.** *Let C be a p×(p+1) matrix such that Cθ=β, i.e.,*

$$C=\left(\begin{array}{ccccc} 0 & 1/{\tilde{\gamma }}_{0} & 0 & \cdots & 0\\ 0 & 0 & 1/{\tilde{\gamma }}_{0} & \cdots & 0\\ \vdots & \vdots & \vdots & \ddots & \vdots \\ 0 & 0 & 0 & \cdots & 1/{\tilde{\gamma }}_{0} \end{array}\right).$$

*Denote $\beta_{0}$ the true value of β. Then, following Theorem 1, we have $\sqrt{N}\left(\hat{\beta }-\beta_{0}\right)\overset{d}{\to }N\left(0,CA^{-1}\Sigma A^{-1}C^{{}^{T}}\right)$.*

**Variance Estimation**. With Theorem 1 and Corollary 1, the variance estimation is straightforward using the plugging in strategy. Note that $var\left(\hat{\theta }\right)=\frac{1}{N}A^{-1}\Sigma A^{-1}$, then one would have the estimate $\hat{var}\left(\hat{\theta }\right)=\frac{1}{N}{\hat{A}}^{-1}\hat{\Sigma }{\hat{A}}^{-1}$ where A^=N2−1∑1≤i<j≤N∂2ϕij(θ^,η^)∂θ∂θT,

$\hat{\Sigma }=\frac{4}{N-1}\sum_{i=1}^{N}{\left[\frac{1}{N-1}\sum_{j=1,j\neq i}^{N}\left\{\hat{B}{\hat{G}}^{-1}M_{ij}\left(\hat{\eta }\right)-N_{ij}\left(\hat{\theta },\hat{\eta }\right)\right\}\right]}^{⊗2}$, B^=N2−1∑1≤i<j≤N∂2ϕij(θ^,η^)∂θ∂ηT, and $\hat{G}=\frac{1}{N}\sum_{i=1}^{N}\frac{\partial^{2}}{\partial \eta \partial \eta^{{}^{T}}}\log\left\{p(z_{i}|u_{i};\hat{\eta })\right\}$.

## 4. Modern Setting with Regularization

In the past few decades, it has become a standard practice to consider the high-dimensional regression model, where one assumes the parameter β is sparse and often uses the regularization technique to recover the sparsity. While it is a prominent problem to analyze this type of model when the data are prone to missing values, the literature is quite scarce primarily because it is cumbersome to rigorously address the missingness under high dimensionality. Therefore, it is valuable to extend the nuisance-free likelihood procedure proposed in Section 3 to the setting with regularization.

**Computation**. Regularization is a powerful technique to identify the zero elements of a sparse parameter in a regression model. Various penalty functions have been extensively studied, such as LASSO [20], SCAD [21], and MCP [22]. In particular, we study the adaptive LASSO penalty [23] with the objective of minimizing the following function

(9) $$\begin{array}{c} L_{\lambda }\left(\theta \right)=L\left(\theta \right)+\sum_{j=1}^{p}\lambda {\left|{\hat{\tilde{\beta }}}_{j}\right|}^{-1}\left|{\tilde{\beta }}_{j}\right|, \end{array}$$

where λ>0 is the tuning parameter. Following [23], ${\hat{\tilde{\beta }}}_{j}$ is a root-*N*-consistent estimator of ${\tilde{\beta }}_{j}$; for example, one can use the estimator via minimizing the unregularized objective Function (6). Obviously, the penalty term in (9) does not alter the numerical characteristic of L(θ) that we presented in Section 3. The $L_{\lambda }\left(\theta \right)$ is essentially the regularized log-likelihood of a logistic regression model with the similar format as discussed in (7).

To choose the tuning parameter λ, one can follow either the cross-validation method or various information-based criteria. Fortunately, all of these approaches have been extensively studied in the literature. In this paper, we follow the Bayesian information criterion (BIC) to determine λ. Specifically, we choose λ to be the minimizer of the following BIC function

$$\begin{array}{c} \mathrm{BIC}\left(\lambda \right)=2L\left(\theta \right)+p_{\lambda }\frac{\log(n)}{n}, \end{array}$$

where $p_{\lambda }$ is the number of nonzero elements in ${\hat{\tilde{\beta }}}_{\lambda }$ and the minimizer of (9) is encoded as ${\hat{\theta }}_{\lambda }={\left({\hat{\tilde{\gamma }}}_{\lambda },{\hat{\tilde{\beta }}}_{\lambda }^{T}\right)}^{T}$. We summarize the whole computation pipeline as Algorithm 2 below.
**Algorithm 2** Minimization of (9) with the ALASSO penalty1: **Inputs:**   $\left\{y_{i},u_{i},z_{i}\right\},\left\{y_{j},u_{j},z_{j}\right\},{\hat{W}}_{ij}$, for i=1,…,n and j=1,…,n 2: **Initialize:**  k←0 3: **for**
j∈{2:n}
**do**
4:  **for**
i∈{1:(j−1)}
**do** 5:    k←k+1 6:    $y_{i\setminus j}\leftarrow y_{i}-y_{j}$, $u_{i\setminus j}\leftarrow u_{i}-u_{j}$, $z_{i\setminus j}\leftarrow z_{i}-z_{j}$, ${\hat{W}}_{k}\leftarrow {\hat{W}}_{ij}$ 7:    $g_{k}\leftarrow I\left\{z_{i\setminus j}>0\right\}$ 8:    $s_{k}\leftarrow -\mathrm{sign}\left(z_{i\setminus j}\right)$ 9:    $t_{k}\leftarrow \left(\right|z_{i\setminus j}|y_{i\setminus j},|z_{i\setminus j}{\left|u_{i\setminus j}^{{}^{T}}\right)}^{{}^{T}}$ 10: Fit logistic regression with response g, covariates t, offset $s^{{}^{T}}\log W$, and no intercept. 11: Obtain $\hat{\tilde{\theta }}$. 12: Fit logistic regression with ALASSO penalty. 13: Find $\lambda^{⋆}$ which minimizes the BIC. 14: **Outputs:**  $\hat{\theta }\left(\lambda^{⋆}\right)={\hat{\theta }}_{\lambda }$


**Asymptotic Theory**. Recall that $\theta ={\left(\tilde{\gamma },{\tilde{\beta }}^{T}\right)}^{T}$. Without loss of generality, we assume the first $p_{0}$ parameters in $\tilde{\beta }$ are nonzero, where $1\leq p_{0}<p$. For simplicity, we denote $\theta_{T}={\left(\tilde{\gamma },{\tilde{\beta }}_{1},\ldots ,{\tilde{\beta }}_{p_{0}}\right)}^{T}$ as the vector of nonzero components and $\theta_{T^{c}}={\left({\tilde{\beta }}_{p_{0}+1},\ldots ,{\tilde{\beta }}_{p}\right)}^{T}$ as the vector of zeros.

In Theorem 1, we defined $A=E\left\{\frac{\partial^{2}\phi_{ij}\left(\theta_{0},\eta_{0}\right)}{\partial \theta \partial \theta^{{}^{T}}}\right\}$, a (p+1)×(p+1) matrix. Now we assume it can be partitioned as $A=\left(\begin{array}{cc} A_{1} & A_{2}\\ A_{2}^{{}^{T}} & A_{3} \end{array}\right)$, where $A_{1}$ is a $\left(p_{0}+1\right)\times \left(p_{0}+1\right)$ submatrix corresponding to $\theta_{T}$. Similarly, we defined $\Sigma =4E\left\{\lambda_{12}\left(\theta_{0},\eta_{0}\right)\lambda_{13}{\left(\theta_{0},\eta_{0}\right)}^{{}^{T}}\right\}$, and we also assume it can be partitioned as $\Sigma =\left(\begin{array}{cc} \Sigma_{1} & \Sigma_{2}\\ \Sigma_{2}^{{}^{T}} & \Sigma_{3} \end{array}\right)$, where $\Sigma_{1}$ is a $\left(p_{0}+1\right)\times \left(p_{0}+1\right)$ submatrix corresponding to $\theta_{T}$ as well. We denote the minimizer of (9), ${\hat{\theta }}_{\lambda }$, as ${\hat{\theta }}_{\lambda }={\left({\hat{\theta }}_{\lambda ,T}^{T},{\hat{\theta }}_{\lambda ,T^{c}}^{T}\right)}^{T}$, and its true value $\theta_{0}={\left(\theta_{0,T}^{T},\theta_{0,T^{c}}^{T}\right)}^{T}$.

Now, we present the oracle property pertaining to ${\hat{\theta }}_{\lambda }$, which includes the asymptotic normality for the nonzero components and the variable selection consistency. The proof is provided in Appendix B.

**Theorem** **2.** *Assume (8), $A_{1}$ is positive definite and $E∥\frac{\partial \phi_{ij}\left(\theta_{0},\eta_{0}\right)}{\partial \theta }{∥}^{2}<\infty$ for each θ in a neighborhood of $\theta_{0}$. We also assume $\sqrt{N}\lambda \to 0$ and Nλ→∞. Then,*

$$\sqrt{N}\left({\hat{\theta }}_{\lambda ,T}-\theta_{0,T}\right)\overset{d}{\to }N\left(0,A_{1}^{-1}\Sigma_{1}A_{1}^{-1}\right).$$

*In addition, let $T_{N}=\left\{j\in \left\{1,\ldots ,p\right\}:{\hat{\tilde{\beta }}}_{j,\lambda }\neq 0\right\}$ and $T=\{j\in \left\{1,\ldots ,p\right\}:{\tilde{\beta }}_{j,0}\neq 0\}$, then*

$$\lim_{N\to \infty }\mathrm{pr}\left(T_{N}=T\right)=1.$$

**Variance Estimation**. Although the above theory provides a rigorous justification for the asymptotic property of ${\hat{\theta }}_{\lambda }$, in practice, however, it does not guide the standard error estimation. Here, we propose a data perturbation approach for the variance estimation. Specifically, following [24], we generate a set of independent and identically distributed positive random variables $\Xi =\{\xi_{i},i=1,\ldots ,N\}$ with $E(\xi_{i})=1$ and $var(\xi_{i})=1$, e.g., the standard exponential distribution. Since it is based on a U-statistic structure, we perturb our objective function by adding $\kappa_{ij}=\xi_{i}\xi_{j}$ to each of its pairwise terms. We first obtain the estimator ${\hat{\theta }}^{⋆}$ by minimizing the perturbed version of (6):

L⋆(θ)=N2−1∑1≤i<j≤Nκijϕij(θ).

Then, we obtain the estimator ${\hat{\theta }}_{\lambda }^{⋆}$ by minimizing the perturbed version of (9):

Lλ⋆(θ)=N2−1∑1≤i<j≤Nκijϕij(θ)+∑j=1pλβ˜^j⋆β˜j,

where the optimal λ is also computed by the BIC. We repeat this data perturbation scheme a large number of times, say, *M*.

Following the theory in [25,26], under some regularity conditions, one can first show that $\sqrt{N}\left({\hat{\theta }}_{\lambda ,T}^{⋆}-\theta_{0,T}\right)$ converges in distribution to $N(0,A_{1}^{-1}\Sigma_{1}A_{1}^{-1})$, the same limiting distribution of $\sqrt{N}\left({\hat{\theta }}_{\lambda }-\theta_{0}\right)$. Furthermore, one can also show ${\mathrm{pr}}^{*}\left({\hat{\theta }}_{\lambda ,T^{c}}^{⋆}=0\right)\to 1$, where ${\mathrm{pr}}^{*}$ is the probability measure generated by the original data X and the perturbation data Ξ. In addition, one can show that the distribution of $\sqrt{N}\left({\hat{\theta }}_{\lambda ,T}^{⋆}-{\hat{\theta }}_{\lambda ,T}\right)$ conditional on the data can be used to approximate the unconditional distribution of $\sqrt{N}\left({\hat{\theta }}_{\lambda ,T}-\theta_{0,T}\right)$ and that ${\mathrm{pr}}^{*}\left({\hat{\theta }}_{\lambda ,T^{c}}^{⋆}=0|X\right)\to 1$.

To achieve a confidence interval for $\theta_{j}$, the *j*-th coordinate in θ, the lower and upper bounds can be formed by ${\hat{\theta }}_{\lambda ,j,\alpha /2}^{⋆}$ and ${\hat{\theta }}_{\lambda ,j,1-\alpha /2}^{⋆}$, respectively, where ${\hat{\theta }}_{\lambda ,j,q}^{⋆}$ represents the *q*-th quantile of $\left\{{\hat{\theta }}_{\lambda ,j,m}^{⋆},m=1,\ldots ,M\right\}$.

## 5. Simulation Studies

We conduct comprehensive simulation studies to evaluate the finite sample performance of our proposed estimators and also compare with some currently existing methods. We first present the results under the model without regularization, then with regularization.

### 5.1. Scenarios without Regularization

For the proposed estimator studied in Section 3, we generate $\{R_{i},Y_{i},U_{i}^{T},Z_{i}\},i=1,\ldots ,N$, independent and identically distributed copies of $\left(R,Y,U^{T},Z\right)$, as follows. We first generate the random vector $U={\left(U_{1},\ldots ,U_{p}\right)}^{T}$ with $U_{i}∼N\left(0.5,1\right)$ and p=4, and then generate $Z=\alpha_{z}+\eta^{T}U+\epsilon_{z}$ with $\alpha_{z}=0.5$, $\eta ={\left(-0.5,1,-1,1.5\right)}^{T}$, $\epsilon_{z}∼N\left(0,1\right)$. Afterwards, the outcome variable *Y* is generated following the model (1) with α=−1, $\beta ={\left(-0.5,1,-1,1.5\right)}^{T}$, γ=0.5, and ϵ∼N(0,1), and the missingness indicator *R* is generated following $\mathrm{pr}\left(R=1|Y,U\right)=I\left(Y<2.5,U_{1}<2,U_{2}<2,U_{3}<2,U_{4}<2\right)$ which results in around 40% missing values. We examine two situations with sample size N=500 and N=1000 respectively. Besides the estimator studied in Section 3 (Proposed), we also implement the estimator using all simulated data (FullData) and the estimator using completely observed subjects only (CC). Based on 1000 simulation replicates, for each of the three estimators, we summarize the sample bias, sample standard deviation, estimated standard error, and coverage probability of 95% confidence intervals in Table 1.

Furthermore, we consider a similar simulation setting where the generation is the same as above except for a logistic missingness mechanism model with $\mathrm{logit}\left\{\mathrm{pr}\left(R=1|Y,U\right)\right\}=3-2Y+0.5U_{1}-U_{2}+U_{3}-1.5U_{4}$, which also results in around 40% missing values. We replicate the results, shown in Table 2.

We can reach the following conclusions from Table 1 and Table 2. For the estimator Proposed, although its bias is slightly larger than the benchmark FullData, it is still very close to zero. The sample standard deviation and the estimated standard error are rather close to each other. The sample coverage probability of the estimated 95% confidence interval is also very close to the nominal level. This observation well matches our theoretical justification in Theorem 1. On the contrary, the estimator CC is clearly biased, resulting in empirical coverage far from the nominal level, and therefore is not recommended to use in practice. It is also clear that, compared to the benchmark FullData, the estimator Proposed has estimation efficiency loss to some extent. This is because the proposed method uses the conditional likelihood approach and it completely eliminates the effect of the nuisance.

### 5.2. Scenarios with Regularization

For the estimator studied in Section 4, the independent and identically distributed samples are generated as follows. The variable $U={\left(U_{1},\ldots ,U_{p}\right)}^{T}$ is generated from $\mathrm{MVN}(0,\Sigma_{u})$ with $\Sigma_{u}={\left(0.5^{\left|i-j\right|}\right)}_{1\leq i,j\leq p}$ and p=8. Then, the shadow variable *Z* is generated following $Z=\alpha_{z}+\eta^{T}U+\epsilon_{z}$ with $\alpha_{z}=0$, $\eta ={\left(-0.5,0.5,-1,1,-0.5,0.5,-1,1\right)}^{T}$ and $\epsilon_{z}∼N\left(0,1\right)$. The outcome variable *Y* is generated from model (1) with α=0, $\beta ={\left(3,1.5,0,0,2,0,0,0\right)}^{T}$, γ=3, $\epsilon ∼N(0,\sigma^{2})$ and σ=3. The distribution of the missingness indicator follows from $\mathrm{logit}\left\{\mathrm{pr}\left(R=1|Y,U\right)\right\}=5+5Y+0.2U_{1}+0.2U_{7}$, which results in about 45% missing values. Similar to Section 5.1, we also examine two situations with sample size N=500 and N=1000 respectively, and we implement three estimators FullData, CC, and Proposed. When the estimator Proposed is implemented, we perform M=500 perturbations in order to obtain the confidence interval for the unknown parameter. The results summarized below are based on 1000 simulation replicates.

**Figure 1:**
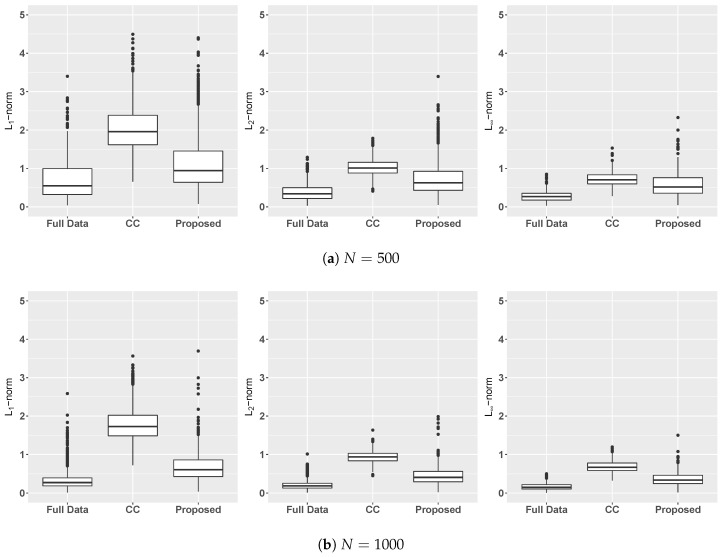
In Section 5.2, $L_{1}$ (1st column), $L_{2}$ (2nd column), and $L_{\infty }$ (3rd column) norms of the estimation bias of the estimator of FullData (using all simulated data), CC (using only completely observed subjects), and of the proposed estimator studied in Section 4.

Figure 1 shows the $L_{1}$, $L_{2}$, and $L_{\infty }$ norms of the bias for the three different estimators. As sample size increases, there is no doubt that the estimation bias is getting smaller for any method. It is also clear that the bias of the Proposed estimator is larger than the benchmark FullData, but much smaller than the method CC.

We present the statistical inference results in Table 3 for N=500 and Table 4 for N=1000, respectively, including sample bias, sample standard deviation, estimated standard error, coverage probability, and length of 95% confidence interval for the three different methods. For the nonzero β’s as well as $\tilde{\gamma }$, similar to Section 5.1, the method CC clearly prompts coverage probability far from the nominal level hence is not reliable. For the method Proposed, its estimation bias is quite close to zero, and its sample standard deviation and estimated standard error are quite close to each other. The coverage probability of the confidence interval converges to the nominal level 95% as the sample size gets larger. For the noisy zero β’s, the coverage probabilities in the three methods are all close to 1, reflecting the variable selection consistency in the oracle property, even for the CC method. Furthermore, a very nice finite sample property of our proposed estimator is that it produces the confidence interval with the shortest length, which can be clearly seen from both Table 3 and Table 4.

## 6. Real Data Application

The Medical Information Mart for Intensive Care III (MIMIC-III) is an openly available electronic health records (EHR) database, developed by the MIT Lab for Computational Physiology [13], comprising de-identified health-related data associated with intensive care unit patients with rich information including demographics, vital signs, laboratory test, medications, and more.

Our initial motivation for this data analysis is to understand the missingness mechanism for some laboratory test biomarkers in this EHR system. As for the EHR database, since the data are collected in a non-prescheduled fashion, i.e., only available when the patient seeks care or the physician orders care, the visiting process could be potentially informative about the patients’ risk categories. Therefore, it is very plausible that the data are missing not at random, or a mix of missing not at random and missing at random [27,28]. When we first conducted the data cleaning process briefly, an interesting phenomenon we observe is that, compared to most biomarkers which usually have <3% missing values, the albumin level in the blood sample, a very indicative biomarker associated with different types of diseases [29], has around 30% missingness.

To further understand this phenomenon, we concentrate on a subset of the data with sample size N=1359 in which 421 samples have missing values in the albumin level but all other variables are complete. We aim to apply the proposed method to the study of the albumin level (*Y*). The calcium level in the blood sample, free of missing data, has been shown in the biomedical literature that it has high correlation with the albumin level [30,31,32]; therefore, we adopt the calcium level as the shadow variable *Z*. Seventeen other variables comprise the vector U, which are either demographics (age and gender), chart events (respiratory rate, glucose, heart rate, systolic blood pressure, diastolic blood pressure, and temperature), other laboratory tests (urea nitrogen, platelets, magnesium, hematocrit, red blood cell, white blood cell, and peripheral capillary oxygen saturation (SpO2)), or aggregated metrics (simplified acute physiology score (SAPS-II) and sequential organ failure assessment score (SOFA)).

**Figure 2:**
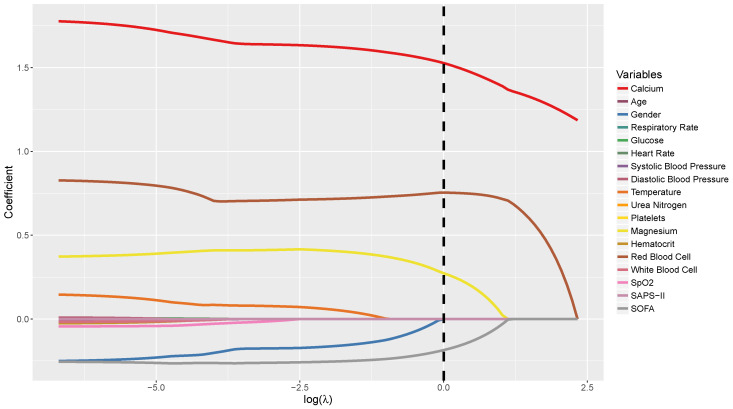
In Section 6, as tuning parameter λ varies, the solution path of the proposed estimator in the MIMIC-III study. The optimal λ, $\lambda^{*}$, equals 1.0030 and $\log\lambda^{*}=0.0030$.

We implement the proposed estimator studied in Section 4 to achieve both variable selection and post-selection inference. We also compare it with the CC method which naively fits the regularized linear regression with the ALASSO penalty. For each of the methods, we apply the data perturbation scheme presented in Section 4 with M=500 for standard error estimation. The results are summarized in Table 5. The solution path of the Proposed method, as the tuning parameter λ varies, is also provided in Figure 2.

In general, both methods achieve the goal of variable selection and post-selection inference by leveraging the regularization technique coupled with the data perturbation strategy, and identify many variables as noise with zero coefficients. In particular, the Proposed method provides larger effects for the calcium level (the shadow variable) and the red blood cell count, whereas a smaller effect for the aggregated SOFA score. The Proposed method simplifies the body temperature and the white blood cell count as nonsignificant variables, which are identified as nonzero but with a very small effect using the CC method. It is also worthwhile to mention that the Proposed method signifies the magnesium level with a quite significant coefficient, which was extensively investigated in the scientific literature [33,34,35].

## 7. Discussion

In this paper, we provide a systematic approach for parameter estimation and statistical inference in both traditional linear model where the regularization is not needed and the modern regularized regression setting, when the outcome variable is prone to missing values and the missingness mechanism can be arbitrarily flexible. A pivotal condition rooted in our procedure is the shadow variable *Z*, which overcomes the model identifiability problem and enables the nuisance-free conditional likelihood process.

Certainly any method would have its own limitations and could be potentially improved. One needs a model p(z|u) to implement the proposed estimator in Section 3 and Section 4. As its modeling does not involve any missing data, we simply use the parametric maximum likelihood estimation in our algorithm as well as in the theoretical justification. Indeed, any statistical or machine learning method can be used for modeling p(z|u). For instance, if one would like to consider a semiparametric model [36], e.g.,

$$\begin{array}{c} p\left(z|u;\eta ,F\right)=\frac{\exp\left(\eta^{T}uz\right)f\left(z\right)}{\int \exp\left(\eta^{T}uz\right)dF\left(z\right)}, \end{array}$$

where $\eta ={\left(\eta_{1},\ldots ,\eta_{p}\right)}^{{}^{T}}$ is a vector of unknown parameters and f(z) is the density of an unknown baseline distribution function *F* with respect to some dominating measure ν. With this model fitted, $W_{ij}$ can be simplified to $W_{ij}=\exp\left(-z_{i\setminus j}\eta^{{}^{T}}u_{i\setminus j}\right)$. Therefore, a similar conditional likelihood approach can be used to estimate η without estimating the nonparametric component f(z).

## Figures and Tables

**Table 1:** In Section 5.1, sample bias (Bias), sample standard deviation (SD), estimated standard error (SE), and coverage probability (CP) of 95% confidence interval of the estimator of FullData (using all simulated data), CC (using only completely observed subjects), and of the proposed estimator studied in Section 3.

*N*	Parameter	Method	Bias	SD	SE	CP
500	$\tilde{\gamma }$	FullData	0.0026	0.0444	0.0450	0.9540
		CC	−0.0329	0.0564	0.0560	0.9100
		Proposed	0.0174	0.0829	0.0789	0.9450
	$\beta_{1}$	FullData	0.0022	0.0489	0.0503	0.9510
		CC	0.0376	0.0670	0.0699	0.9300
		Proposed	0.0164	0.1644	0.1607	0.9400
	$\beta_{2}$	FullData	−0.0017	0.0657	0.0635	0.9310
		CC	−0.0649	0.0851	0.0835	0.8680
		Proposed	−0.0399	0.2305	0.2239	0.9360
	$\beta_{3}$	FullData	0.0022	0.0616	0.0635	0.9540
		CC	0.0778	0.0871	0.0867	0.8430
		Proposed	0.0462	0.2323	0.2298	0.9410
	$\beta_{4}$	FullData	−0.0045	0.0792	0.0810	0.9530
		CC	−0.0988	0.1007	0.1043	0.8550
		Proposed	−0.0672	0.3081	0.3047	0.9380
1000	$\tilde{\gamma }$	FullData	−0.0012	0.0317	0.0317	0.9540
		CC	−0.0348	0.0396	0.0393	0.8510
		Proposed	0.0068	0.0573	0.0555	0.9350
	$\beta_{1}$	FullData	0.0011	0.0367	0.0355	0.9370
		CC	0.0399	0.0490	0.0494	0.8840
		Proposed	0.0154	0.1154	0.1138	0.9460
	$\beta_{2}$	Full Data	0.0020	0.0448	0.0448	0.9500
		CC	−0.0649	0.0577	0.0588	0.8110
		Proposed	−0.0153	0.1531	0.1591	0.9590
	$\beta_{3}$	Full Data	−0.0015	0.0458	0.0449	0.9460
		CC	0.0779	0.0605	0.0611	0.7490
		Proposed	0.0135	0.1598	0.1634	0.9480
	$\beta_{4}$	Full Data	0.0009	0.0564	0.0571	0.9540
		CC	−0.0949	0.0720	0.0734	0.7550
		Proposed	−0.0242	0.2091	0.2167	0.9430

**Table 2:** In Section 5.1, sample bias (Bias), sample standard deviation (SD), estimated standard error (SE), and coverage probability (CP) of 95% confidence interval of the estimator of FullData (using all simulated data), CC (using only completely observed subjects), and of the proposed estimator studied in Section 3, with a logistic missingness mechanism model.

*N*	Parameter	Method	Bias	SD	SE	CP
500	$\tilde{\gamma }$	FullData	−0.0011	0.0464	0.0451	0.9410
		CC	−0.0306	0.0567	0.0567	0.9200
		Proposed	0.0100	0.0822	0.0787	0.9380
	$\beta_{1}$	FullData	−0.0004	0.0509	0.0503	0.9520
		CC	0.0440	0.0636	0.0637	0.8930
		Proposed	0.0146	0.1308	0.1236	0.9420
	$\beta_{2}$	FullData	0.0013	0.0639	0.0637	0.9520
		CC	−0.0871	0.0828	0.0821	0.8190
		Proposed	−0.0173	0.1824	0.1753	0.9430
	$\beta_{3}$	FullData	−0.0030	0.0655	0.0636	0.9400
		CC	0.0876	0.0847	0.0821	0.8030
		Proposed	0.0214	0.1840	0.1756	0.9440
	$\beta_{4}$	FullData	0.0023	0.0845	0.0812	0.9390
		CC	−0.1307	0.1083	0.1061	0.7560
		Proposed	−0.0331	0.2533	0.2384	0.9360
1000	$\tilde{\gamma }$	FullData	0.0004	0.0315	0.0317	0.9490
		CC	−0.0286	0.0396	0.0398	0.8950
		Proposed	0.0060	0.0568	0.0555	0.9390
	$\beta_{1}$	FullData	0.0007	0.0362	0.0354	0.9420
		CC	0.0442	0.0451	0.0447	0.8410
		Proposed	0.0079	0.0910	0.0859	0.9290
	$\beta_{2}$	FullData	−0.0004	0.0450	0.0448	0.9390
		CC	−0.0879	0.0571	0.0576	0.6640
		Proposed	−0.0044	0.1277	0.1220	0.9420
	$\beta_{3}$	FullData	−0.0009	0.0450	0.0448	0.9450
		CC	0.0880	0.0588	0.0577	0.6660
		Proposed	0.0114	0.1309	0.1222	0.9380
	$\beta_{4}$	FullData	−0.0005	0.0576	0.0572	0.9510
		CC	−0.1342	0.0755	0.0745	0.5740
		Proposed	−0.0191	0.1757	0.1661	0.9370

**Table 3:** In Section 5.2, with sample size N=500, sample bias (Bias), sample standard deviation (SD), estimated standard error (SE), coverage probability (CP), and length (Length) of 95% confidence interval of the estimator of FullData (using all simulated data), CC (using only completely observed subjects) and of the proposed estimator studied in Section 4.

Parameter		Method	Bias	SD	SE	CP	Length
$\tilde{\gamma }$		FullData	0.0001	0.0120	0.0132	0.9480	0.0515
		CC	−0.0729	0.0180	0.0183	0.0370	0.0716
		Proposed	−0.0423	0.0500	0.0498	0.8200	0.1926
True Nonzero	$\beta_{1}$	FullData	0.0021	0.1686	0.1649	0.9400	0.6415
		CC	−0.6547	0.2207	0.2114	0.1460	0.8233
		Proposed	0.0354	0.4698	0.4746	0.9320	1.8513
	$\beta_{2}$	Full Data	−0.0275	0.1692	0.1791	0.9440	0.6952
		CC	−0.3501	0.2227	0.2174	0.6180	0.8471
		Proposed	−0.2654	0.5843	0.5609	0.8940	1.9237
	$\beta_{5}$	Full Data	−0.0172	0.1576	0.1756	0.9650	0.6826
		CC	−0.4478	0.2172	0.2161	0.4370	0.8418
		Proposed	−0.1251	0.4037	0.4611	0.9330	1.8063
True Zero	$\beta_{3}$	FullData	0.0085	0.1567	0.1890	0.9960	0.7184
		CC	0.0063	0.2067	0.2304	0.9890	0.8890
		Proposed	0.0109	0.0988	0.1690	1.0000	0.4398
	$\beta_{4}$	Full Data	−0.0019	0.1581	0.1900	0.9940	0.7206
		CC	−0.0017	0.2097	0.2307	0.9900	0.8914
		Proposed	0.0126	0.1112	0.1447	1.0000	0.3668
	$\beta_{6}$	Full Data	0.0045	0.1212	0.1606	0.9980	0.6146
		CC	−0.0053	0.1749	0.1953	0.9900	0.7560
		Proposed	0.0034	0.0664	0.1160	1.0000	0.2555
	$\beta_{7}$	Full Data	0.0014	0.1351	0.1839	0.9980	0.7063
		CC	−0.0055	0.1870	0.2245	0.9950	0.8717
		Proposed	0.0024	0.0386	0.1115	1.0000	0.2538
	$\beta_{8}$	Full Data	−0.0072	0.1295	0.1748	0.9990	0.6653
		CC	−0.0062	0.1795	0.2125	0.9940	0.8251
		Proposed	0.0016	0.0741	0.1066	1.0000	0.2284

**Table 4:** In Section 5.2, with sample size N=1000, sample bias (Bias), sample standard derivation (SD), estimated standard error (SE), coverage probability (CP), and length (Length) of 95% confidence interval of the estimator of FullData (using all simulated data), CC (using only completely observed subjects) and of the proposed estimator studied in Section 4.

Parameter		Method	Bias	SD	SE	CP	Length
$\tilde{\gamma }$		FullData	−0.0005	0.0073	0.0088	0.9690	0.0344
		CC	−0.0730	0.0126	0.0130	0.0000	0.0507
		Proposed	−0.0213	0.0311	0.0334	0.8700	0.1293
True Nonzero	$\beta_{1}$	FullData	−0.0005	0.1186	0.1170	0.9300	0.4547
		CC	−0.6655	0.1568	0.1507	0.0090	0.5864
		Proposed	0.0211	0.2911	0.2969	0.9300	1.1631
	$\beta_{2}$	Full Data	−0.0321	0.1175	0.1249	0.9550	0.4861
		CC	−0.3387	0.1477	0.1534	0.3960	0.5972
		Proposed	−0.0979	0.2907	0.3383	0.9230	1.3115
	$\beta_{5}$	Full Data	−0.0225	0.1051	0.1206	0.9590	0.4698
		CC	−0.4485	0.1478	0.1534	0.1770	0.5964
		Proposed	−0.0621	0.2351	0.2526	0.9290	0.9871
True Zero	$\beta_{3}$	FullData	−0.0007	0.0621	0.1162	1.0000	0.4253
		CC	0.0023	0.1414	0.1614	0.9920	0.6180
		Proposed	0.0044	0.0581	0.0910	1.0000	0.2091
	$\beta_{4}$	Full Data	0.0020	0.0632	0.1170	1.0000	0.4271
		CC	−0.0005	0.1333	0.1608	0.9930	0.6207
		Proposed	0.0063	0.0584	0.0887	1.0000	0.2107
	$\beta_{6}$	Full Data	0.0013	0.0571	0.1010	1.0000	0.3670
		CC	−0.0034	0.1159	0.1378	0.9950	0.5313
		Proposed	0.0012	0.0281	0.0688	1.0000	0.1430
	$\beta_{7}$	Full Data	−0.0028	0.0599	0.1144	1.0000	0.4231
		CC	−0.0033	0.1243	0.1584	0.9970	0.6131
		Proposed	0.0016	0.0288	0.0698	1.0000	0.1421
	$\beta_{8}$	Full Data	0.0039	0.0589	0.1080	1.0000	0.3970
		CC	0.0028	0.1256	0.1497	0.9940	0.5752
		Proposed	0.0000	0.0333	0.0644	1.0000	0.1314

**Table 5:** In Section 6, the parameter estimate (Estimate), standard error (SE), and confidence interval (CI) of the estimator of CC (using only completely observed subjects) and of the proposed estimator studied in Section 4 in the MIMIC−III study.

Effect	CC			Proposed		
	Estimate	SE	CI	Estimate	SE	CI
Calcium(shadow)	0.7707	0.0691	[0.6532, 0.9153]	1.5271	0.1796	[1.1815, 1.8835]
Red Blood Cell	0.6491	0.0514	[0.5337, 0.7257]	0.7545	0.1631	[0.3594, 1.0109]
Magnesium	0.0000	0.0686	[−0.2073, 0.0000]	0.2731	0.2452	[0.0000, 0.6609]
SOFA	−0.2720	0.0268	[−0.3135, −0.2099]	−0.1852	0.1040	[−0.3467, 0.0000]
Temperature	−0.0360	0.0351	[−0.0883, 0.0659]	0.0000	0.0964	[0.0000, 0.3132]
White Blood Cell	−0.0245	0.0123	[−0.0416, 0.0000]	0.0000	0.0025	[0.0000, 0.0000]
Age	0.0000	0.0008	[0.0000, 0.0000]	0.0000	0.0017	[0.0000. 0.0000]
Gender	0.0000	0.0240	[−0.0477, 0.0662]	0.0000	0.1320	[−0.4025, 0.0000]
Respiratory Rate	0.0000	0.0034	[−0.0141, 0.0000]	0.0000	0.0008	[0.0000, 0.0000]
Glucose	0.0000	0.0000	[0.0000, 0.0000]	0.0000	0.0005	[0.0000, 0.0000]
Heart Rate	0.0000	0.0025	[−0.0091, 0.0000]	0.0000	0.0004	[0.0000, 0.0000]
Systolic BP	0.0000	0.0045	[−0.0139, 0.0000]	0.0000	0.0000	[0.0000, 0.0000]
Diastolic BP	0.0000	0.0072	[0.0000, 0.0223]	0.0000	0.0000	[0.0000, 0.0000]
Urea Nitrogen	0.0000	0.0004	[0.0000, 0.0000]	0.0000	0.0000	[0.0000, 0.0000]
Platelets	0.0000	0.0000	[0.0000, 0.0000]	0.0000	0.0000	[0.0000, 0.0000]
Hematocrit	0.0000	0.0027	[0.0000, 0.0000]	0.0000	0.0000	[0.0000, 0.0000]
SpO2	0.0000	0.0145	[−0.0479, 0.0000]	0.0000	0.0162	[0.0000, 0.0000]
SAPS-II	0.0000	0.0106	[−0.0051, 0.0269]	0.0000	0.0000	[0.0000, 0.0000]

## Appendix A. Proof of Theorem 1

**Proof.** Note that $\hat{\theta }$ is obtained by setting estimating equation $\frac{\partial L(\hat{\theta },\hat{\eta })}{\partial \theta }=0$, which is equivalent to

(A1) $$\left\{\frac{\partial L(\hat{\theta },\hat{\eta })}{\partial \theta }-\frac{\partial L(\theta_{0},\hat{\eta })}{\partial \theta }\right\}+\left\{\frac{\partial L(\theta_{0},\hat{\eta })}{\partial \theta }-\frac{\partial L(\theta_{0},\eta_{0})}{\partial \theta }\right\}+\frac{\partial L(\theta_{0},\eta_{0})}{\partial \theta }=0.$$

Specifically,

(A2) $$\frac{\partial L(\hat{\theta },\hat{\eta })}{\partial \theta }-\frac{\partial L(\theta_{0},\hat{\eta })}{\partial \theta }=\frac{\partial^{2}L\left(\theta_{0},\hat{\eta }\right)}{\partial \theta \partial \theta^{{}^{T}}}\left(\hat{\theta }-\theta_{0}\right)+o_{p}\left(N^{-\frac{1}{2}}\right),$$

by Taylor expansion. Similarly,

(A3) $$\frac{\partial L(\theta_{0},\hat{\eta })}{\partial \theta }-\frac{\partial L(\theta_{0},\eta_{0})}{\partial \theta }=\frac{\partial^{2}L\left(\theta_{0},\eta_{0}\right)}{\partial \theta \partial \eta^{{}^{T}}}\left(\hat{\eta }-\eta_{0}\right)+o_{p}\left(N^{-\frac{1}{2}}\right).$$

With (A2) and (A3) plugging into (A1), we can obtain the following equation,

(A4) $$\sqrt{N}\frac{\partial^{2}L\left(\theta_{0},\hat{\eta }\right)}{\partial \theta \partial \theta^{{}^{T}}}\left(\hat{\theta }-\theta_{0}\right)+\sqrt{N}\frac{\partial^{2}L\left(\theta_{0},\eta_{0}\right)}{\partial \theta \partial \eta^{{}^{T}}}\left(\hat{\eta }-\eta_{0}\right)+\sqrt{N}\frac{\partial L(\theta_{0},\eta_{0})}{\partial \theta }+o_{p}\left(1\right)=0.$$

As $\sqrt{N}\left(\hat{\eta }-\eta_{0}\right)=-G^{-1}\sqrt{N}\frac{1}{N}\sum_{i=1}^{N}\frac{\partial }{\partial \eta }\log\left\{p(z_{i}|u_{i};\eta_{0})\right\}+o_{p}\left(1\right)$ from the asymptotic property of $\hat{\eta }$, (A4) is equivalent to

$$\begin{array}{ccc} & & \sqrt{N}\frac{\partial^{2}L\left(\theta_{0},\hat{\eta }\right)}{\partial \theta \partial \theta^{{}^{T}}}\left(\hat{\theta }-\theta_{0}\right)+\frac{\partial^{2}L\left(\theta_{0},\eta_{0}\right)}{\partial \theta \partial \eta^{{}^{T}}}\left[-G^{-1}\sqrt{N}\frac{1}{N}\sum_{i=1}^{N}\frac{\partial }{\partial \eta }\log\left\{p(z_{i}|u_{i};\eta_{0})\right\}\right]\\ & + & \sqrt{N}\frac{\partial L(\theta_{0},\eta_{0})}{\partial \theta }+o_{p}\left(1\right)=0. \end{array}$$

Thus,

(A5) $$\begin{array}{ccc} & & \sqrt{N}\left(\hat{\theta }-\theta_{0}\right)\\ & = & -{\left\{\frac{\partial^{2}L\left(\theta_{0},\hat{\eta }\right)}{\partial \theta \partial \theta^{{}^{T}}}\right\}}^{-1}\times \left\{\frac{\partial^{2}L\left(\theta_{0},\eta_{0}\right)}{\partial \theta \partial \eta^{{}^{T}}}\left[-G^{-1}\sqrt{N}\frac{1}{N}\sum_{i=1}^{N}\frac{\partial }{\partial \eta }\log\left\{p(z_{i}|u_{i};\eta_{0})\right\}\right]+\sqrt{N}\frac{\partial L(\theta_{0},\eta_{0})}{\partial \theta }\right\}\\ & & +o_{p}\left(1\right)\\ & = & -A^{-1}\left\{B\left[-G^{-1}\sqrt{N}\frac{1}{N}\sum_{i=1}^{N}\frac{\partial }{\partial \eta }\log\left\{p(z_{i}|u_{i};\eta_{0})\right\}\right]+\sqrt{N}\frac{\partial L(\theta_{0},\eta_{0})}{\partial \theta }\right\}+o_{p}\left(1\right), \end{array}$$

where $\frac{\partial^{2}L\left(\theta_{0},\eta_{0}\right)}{\partial \theta \partial \theta^{{}^{T}}}\overset{p}{\to }A=E\left\{\frac{\partial^{2}\phi_{ij}\left(\theta_{0},\eta_{0}\right)}{\partial \theta \partial \theta^{{}^{T}}}\right\}$, and $\frac{\partial^{2}L\left(\theta_{0},\eta_{0}\right)}{\partial \theta \partial \eta^{{}^{T}}}\overset{p}{\to }B=E\left\{\frac{\partial^{2}\phi_{ij}\left(\theta_{0},\eta_{0}\right)}{\partial \theta \partial \eta^{{}^{T}}}\right\}$. In addition, we need to form a projection of $\frac{1}{N}\sum_{i=1}^{N}\frac{\partial }{\partial \eta }\log\left\{p(z_{i}|u_{i};\eta_{0})\right\}$ in (A5) through

1N∑i=1N∂∂ηlogp(zi|ui;η0)=N2−1∑1≤i<j≤N12∂∂ηlogp(zi|ui;η0)+∂∂ηlogp(zj|uj;η0),

and

∂L(θ0,η0)∂θ=N2−1∑1≤i<j≤N∂ϕij(θ0,η0)∂θ.

To sum up, (A5) can be formed as

Nθ^−θ0=A−1NN2−1∑1≤i<j≤NBG−1Mij(η0)−Nij(θ0,η0)+op(1),

where $M_{ij}\left(\eta_{0}\right)=\frac{1}{2}\left[\frac{\partial }{\partial \eta }\log\left\{p(z_{i}|u_{i};\eta_{0})\right\}+\frac{\partial }{\partial \eta }\log\left\{p(z_{j}|u_{j};\eta_{0})\right\}\right]$ and $N_{ij}\left(\theta_{0},\eta_{0}\right)=\frac{\partial \phi_{ij}\left(\theta_{0},\eta_{0}\right)}{\partial \theta }$.  □

## Appendix B. Proof of Theorem 2

**Proof.** Define function

(A6) $$q_{ij}\left(\theta \right)=\phi_{ij}\left(\theta_{0}+\frac{\theta }{\sqrt{N}},\hat{\eta }\right)-\phi_{ij}\left(\theta_{0},\hat{\eta }\right)-{\left(\frac{\theta }{\sqrt{N}}\right)}^{{}^{T}}\frac{\partial \phi_{ij}\left(\theta_{0},\hat{\eta }\right)}{\partial \theta }=O_{p}\left(\frac{1}{N}\right),$$

and we can form a U-statistic based on $q_{ij}\left(\theta \right)$ as

$$\begin{array}{cc} Q_{N}\left(\theta \right) & =\frac{2}{N(N-1)}\sum_{1\leq i<j\leq N}q_{ij}\left(\theta \right)\\ & =L\left(\theta_{0}+\frac{\theta }{\sqrt{N}}\right)-L\left(\theta_{0}\right)-\frac{1}{\sqrt{N}}\;\cdot \;\frac{2}{N(N-1)}\theta^{{}^{T}}\sum_{1\leq i<j\leq N}\frac{\partial \phi_{ij}\left(\theta_{0},\hat{\eta }\right)}{\partial \theta }. \end{array}$$

The variance of $Q_{N}\left(\theta \right)$ is bounded by $var\left\{Q_{N}\left(\theta \right)\right\}\leq \frac{2}{N}var\left\{q_{ij}\left(\theta \right)\right\}$, from Corollary 3.2 of [37]. Meanwhile, $\frac{2}{N}var\left\{q_{ij}\left(\theta \right)\right\}=\frac{2}{N}\left[E\left\{q_{ij}{\left(\theta \right)}^{2}\right\}-E{\left\{q_{ij}\left(\theta \right)\right\}}^{2}\right]\leq \frac{2}{N}E\left\{q_{ij}{\left(\theta \right)}^{2}\right\}$, as $E{\left\{q_{ij}\left(\theta \right)\right\}}^{2}\geq 0$. As $\phi_{ij}\left(\theta ,\hat{\eta }\right)$ is convex, that is, differentiable at $\theta_{0}$, we can conclude

(A7) $$\phi_{ij}\left(\theta_{0}+\frac{\theta }{\sqrt{N}},\hat{\eta }\right)-\phi_{ij}\left(\theta_{0},\hat{\eta }\right)\geq {\left(\frac{\theta }{\sqrt{N}}\right)}^{{}^{T}}\frac{\partial \phi_{ij}\left(\theta_{0},\hat{\eta }\right)}{\partial \theta },$$

from which we can obtain $q_{ij}\left(\theta \right)\geq 0$. Similarly,

(A8) $$\phi_{ij}\left(\theta_{0}+\frac{\theta }{\sqrt{N}},\hat{\eta }\right)-\phi_{ij}\left(\theta_{0},\hat{\eta }\right)\leq {\left(\frac{\theta }{\sqrt{N}}\right)}^{{}^{T}}\frac{\partial \phi_{ij}\left(\theta_{0}+\frac{\theta }{\sqrt{N}},\hat{\eta }\right)}{\partial \theta }.$$

From (A6)–(A8), we can conclude

$$0\leq q_{ij}\left(\theta \right)\leq {\left(\frac{\theta }{\sqrt{N}}\right)}^{{}^{T}}\left\{\frac{\partial \phi_{ij}\left(\theta_{0}+\frac{\theta }{\sqrt{N}},\hat{\eta }\right)}{\partial \theta }-\frac{\partial \phi_{ij}\left(\theta_{0},\hat{\eta }\right)}{\partial \theta }\right\}.$$

Therefore, we can bound

$$\frac{2}{N}E\left\{q_{ij}{\left(\theta \right)}^{2}\right\}\leq \frac{2}{N}{\left(\frac{1}{\sqrt{N}}\right)}^{2}E{\left[\theta^{{}^{T}}\left\{\frac{\partial }{\partial \theta }\phi_{ij}\left(\theta_{0}+\frac{\theta }{\sqrt{N}},\hat{\eta }\right)-\frac{\partial \phi_{ij}\left(\theta_{0},\hat{\eta }\right)}{\partial \theta }\right\}\right]}^{2}.$$

The term $\theta^{{}^{T}}\left\{\frac{\partial }{\partial \theta }\phi_{ij}\left(\theta_{0}+\frac{\theta }{\sqrt{N}},\hat{\eta }\right)-\frac{\partial \phi_{ij}\left(\theta_{0},\hat{\eta }\right)}{\partial \theta }\right\}\overset{p}{\to }0$ as N→∞. Thus, $var\left\{N\;\cdot \;Q_{N}\left(\theta \right)\right\}\overset{p}{\to }0$ and consequently

(A9) $$N\cdot Q_{N}\left(\theta \right)-N\cdot E\left\{Q_{N}\left(\theta \right)\right\}\overset{p}{\to }0.$$

Meanwhile, $E\left\{Q_{N}\left(\theta \right)\right\}=E\left\{\phi_{ij}\left(\theta_{0}+\frac{\theta }{\sqrt{N}},\hat{\eta }\right)\right\}-E\left\{\phi_{ij}\left(\theta_{0},\hat{\eta }\right)\right\}$. Eventually from (A9) we have

(A10) $$\begin{array}{c} N\left\{L\left(\theta_{0}+\frac{\theta }{\sqrt{N}}\right)-L\left(\theta_{0}\right)\right\}-\theta^{{}^{T}}\sqrt{N}\frac{2}{N(N-1)}\sum_{1\leq i<j\leq N}\frac{\partial \phi_{ij}\left(\theta_{0},\hat{\eta }\right)}{\partial \theta }\\ \;-N\left[E\left\{\phi_{ij}\left(\theta_{0}+\frac{\theta }{\sqrt{N}},\hat{\eta }\right)\right\}-E\left\{\phi_{ij}\left(\theta_{0},\hat{\eta }\right)\right\}\right]\overset{p}{\to }0. \end{array}$$

The third term on the left side of (A10) has convergence properties

$$\begin{array}{ccc} & & N\left[E\left\{\phi_{ij}\left(\theta_{0}+\frac{\theta }{\sqrt{N}},\hat{\eta }\right)\right\}-E\left\{\phi_{ij}\left(\theta_{0},\hat{\eta }\right)\right\}\right]\\ & = & N\left[E\left\{\phi_{ij}\left(\theta_{0},\hat{\eta }\right)+{\left(\frac{\theta }{\sqrt{N}}\right)}^{{}^{T}}\frac{\partial \phi_{ij}\left(\theta_{0},\hat{\eta }\right)}{\partial \theta }+\frac{1}{2}{\left(\frac{\theta }{\sqrt{N}}\right)}^{{}^{T}}\frac{\partial^{2}\phi_{ij}\left(\theta_{0},\hat{\eta }\right)}{\partial \theta \partial \theta^{{}^{T}}}\frac{\theta }{\sqrt{N}}+o_{p}\left(\frac{1}{N}\right)\right\}\right.\\ & & \left.-E\left\{\phi_{ij}\left(\theta_{0},\hat{\eta }\right)\right\}\right]\\ & \overset{p}{\to } & \frac{1}{2}\theta^{{}^{T}}A\theta . \end{array}$$

By CLT for U-statistics,

$$\sqrt{N}\left[\frac{2}{N(N-1)}\sum_{1\leq i<j\leq N}\frac{\partial \phi_{ij}\left(\theta_{0},\hat{\eta }\right)}{\partial \theta }\right]\overset{d}{\to }N\left(0,\Sigma \right).$$

Using Slutsky’s theorem, we can simplify (A10) as

$$N\left\{L\left(\theta_{0}+\frac{\theta }{\sqrt{N}}\right)-L\left(\theta_{0}\right)\right\}\overset{d}{\to }\frac{1}{2}\theta^{{}^{T}}A\theta +\theta^{{}^{T}}W,$$

where W∼N(0,Σ). Based on convexity [38], for every compact set $K\subset R^{p+1}$, we have

(A11) $$\left[N\left\{L\left(\theta_{0}+\frac{\theta }{\sqrt{N}},\hat{\eta }\right)-L\left(\theta_{0},\hat{\eta }\right)\right\}:\theta \in K\right]\overset{d}{\to }\left\{\frac{1}{2}\theta^{{}^{T}}A\theta +\theta^{{}^{T}}W:\theta \in K\right\}.$$

Now we develop large sample properties on the penalty term in objective function with adaptive LASSO penalty. We modify the penalty term as

$$N\sum_{j=1}^{p}\frac{\lambda }{\left|{\hat{\tilde{\beta }}}_{j}\right|}\left|{\tilde{\beta }}_{j,0}+\frac{{\tilde{\beta }}_{j}}{\sqrt{N}}\right|-N\sum_{j=1}^{p}\frac{\lambda }{\left|{\hat{\tilde{\beta }}}_{j}\right|}\left|{\tilde{\beta }}_{j,0}\right|.$$

From Theorem 1, we have already obtained $\sqrt{N}\left({\hat{\tilde{\beta }}}_{j}-{\tilde{\beta }}_{j,0}\right)=O_{p}\left(1\right)$. Meanwhile, Nλ→∞ and $\sqrt{N}\lambda \to 0$. If ${\tilde{\beta }}_{j,0}\neq 0$, then $\sqrt{N}\lambda /\left|{\hat{\tilde{\beta }}}_{j}\right|\overset{p}{\to }0$ and $\left|\sqrt{N}{\tilde{\beta }}_{j,0}+{\tilde{\beta }}_{j}\right|-\left|\sqrt{N}{\tilde{\beta }}_{j,0}\right|\to \mathrm{sign}\left({\tilde{\beta }}_{j,0}\right){\tilde{\beta }}_{j}$. Eventually

$$N\frac{\lambda }{\left|{\hat{\tilde{\beta }}}_{j}\right|}\left(\left|{\tilde{\beta }}_{j,0}+\frac{{\tilde{\beta }}_{j}}{\sqrt{N}}\right|-\left|{\tilde{\beta }}_{j,0}\right|\right)=\sqrt{N}\frac{\lambda }{\left|{\hat{\tilde{\beta }}}_{j}\right|}\left(\left|\sqrt{N}{\tilde{\beta }}_{j,0}+{\tilde{\beta }}_{j}\right|-\left|\sqrt{N}{\tilde{\beta }}_{j,0}\right|\right)\overset{p}{\to }0.$$

If ${\tilde{\beta }}_{j,0}=0$, then $\sqrt{N}\lambda /\left|{\hat{\tilde{\beta }}}_{j}\right|=N\lambda /\left(\sqrt{N}\left|{\hat{\tilde{\beta }}}_{j}\right|\right)\overset{p}{\to }\infty$, consequently

$$N\frac{\lambda }{\left|{\hat{\tilde{\beta }}}_{j}\right|}\left(\left|{\tilde{\beta }}_{j,0}+\frac{{\tilde{\beta }}_{j}}{\sqrt{N}}\right|-\left|{\tilde{\beta }}_{j,0}\right|\right)=\sqrt{N}\frac{\lambda }{\left|{\hat{\tilde{\beta }}}_{j}\right|}\left|{\tilde{\beta }}_{j}\right|\overset{p}{\to }\{\begin{array}{cc} 0, & \mathrm{if}\;{\tilde{\beta }}_{j}=0,\\ \infty , & \mathrm{if}\;{\tilde{\beta }}_{j}\neq 0. \end{array}$$

Therefore, we can summarize

$$N\sum_{j=1}^{p}\frac{\lambda }{\left|{\hat{\tilde{\beta }}}_{j}\right|}\left(\left|{\tilde{\beta }}_{j,0}+\frac{{\tilde{\beta }}_{j}}{\sqrt{N}}\right|-\left|{\tilde{\beta }}_{j,0}\right|\right)\overset{p}{\to }\{\begin{array}{cc} 0, & \mathrm{if}\;\tilde{\beta }=\left({\tilde{\beta }}_{1},\ldots ,{\tilde{\beta }}_{p_{0}},0,\ldots ,0\right),\\ \infty , & \mathrm{otherwise}. \end{array}$$

We have infinity in the limit function, so we cannot use standard argumentation relating to uniform convergence in probability on compacts [39]. However, we can apply slightly more complicated epi-convergence. Thus, based on the works in [23,40,41], we have

(A12) $$N\left\{L\left(\theta_{0}+\frac{\theta }{\sqrt{N}}\right)-L\left(\theta_{0}\right)\right\}+N\sum_{j=1}^{p}\frac{\lambda }{\left|{\hat{\tilde{\beta }}}_{j}\right|}\left(\left|{\tilde{\beta }}_{j,0}+\frac{{\tilde{\beta }}_{j}}{\sqrt{N}}\right|-\left|{\tilde{\beta }}_{j,0}\right|\right)\overset{e-d}{\to }V\left(\theta \right),$$

and

$$V\left(\theta \right)=\{\begin{array}{cc} \frac{1}{2}\theta_{T}^{{}^{T}}A_{1}\theta_{T}+\theta_{T}^{{}^{T}}W_{T}, & \mathrm{if}\;\theta =(\tilde{\gamma },{\tilde{\beta }}_{1},\ldots ,{\tilde{\beta }}_{p_{0}},0,\ldots ,0),\\ \infty , & \mathrm{otherwise}. \end{array}$$

and $W_{T}∼N\left(0,\Sigma_{1}\right)$. Specifically, the left side of (A12) is minimized if $\theta =\sqrt{N}\left({\hat{\theta }}_{\lambda }-\theta_{0}\right)$ and V(θ) has a unique minimizer ${\left(-{\left(A_{1}^{-1}W_{T}\right)}^{{}^{T}},0^{{}^{T}}\right)}^{{}^{T}}$ by setting $\frac{\partial V(\theta )}{\partial \theta }=0$. Therefore, convergence of minimizers [40] can be concluded from (A12):

(A13) $$\sqrt{N}\left({\hat{\theta }}_{\lambda ,T}-\theta_{0,T}\right)\overset{d}{\to }-A_{1}^{-1}W_{T}\;\mathrm{and}\;\sqrt{N}\left({\hat{\theta }}_{\lambda ,T^{c}}-\theta_{0,T^{c}}\right)\overset{d}{\to }0.$$

For j∈T,

$$\mathrm{pr}\left(j\notin T_{N}\right)=\mathrm{pr}\left({\hat{\tilde{\beta }}}_{j,\lambda }=0\right)\to 0.$$

Thus, $\mathrm{pr}\left(T\subset T_{N}\right)\to 1$. In addition, ${\hat{\theta }}_{\lambda }$ minimizes the convex objective function $L_{\lambda }\left(\theta \right)$ so that $0\in \partial L_{\lambda }\left({\hat{\theta }}_{\lambda }\right)$. As $L_{\lambda }\left(\theta \right)$ might be nondifferentiable and gradient of $L_{\lambda }\left(\theta \right)$ does not exist for some θ, we use $\partial L_{\lambda }\left(\theta \right)$ to represent an arbitrary selection of the subgradient of $L_{\lambda }\left(\theta \right)$. By taking the subgradient of the objective function with adaptive LASSO penalty, we can obtain

$$\partial L_{\lambda }\left({\hat{\theta }}_{\lambda }\right)=\partial L\left({\hat{\theta }}_{\lambda }\right)+\partial \left(\sum_{j=1}^{p}\frac{\lambda }{\left|{\hat{\tilde{\beta }}}_{j}\right|}\left|{\hat{\tilde{\beta }}}_{j,\lambda }\right|\right).$$

For j∉T, $\mathrm{pr}\left(j\in T_{N}\right)$ can be upper bounded by

(A14) $$\mathrm{pr}\left(\partial_{j}L\left({\hat{\theta }}_{\lambda }\right)+\frac{\lambda }{\left|{\hat{\tilde{\beta }}}_{j}\right|}\mathrm{sign}\left({\hat{\tilde{\beta }}}_{j,\lambda }\right)=0\right)\leq \mathrm{pr}\left(\sqrt{N}\left|\partial_{j}L\left({\hat{\theta }}_{\lambda }\right)\right|=\sqrt{N}\frac{\lambda }{\left|{\hat{\tilde{\beta }}}_{j}\right|}\right),$$

where $\partial_{j}$ is the *j*-th coordinate of subgradient and $\sqrt{N}\lambda /\left|{\hat{\tilde{\beta }}}_{j}\right|\overset{p}{\to }\infty$ as j∉T. We can expand the subgradient $\sqrt{N}\partial L\left({\hat{\theta }}_{\lambda }\right)$ as

(A15) $$\sqrt{N}\partial L\left({\hat{\theta }}_{\lambda }\right)=\sqrt{N}\left\{\partial L\left({\hat{\theta }}_{\lambda }\right)-\partial L\left(\theta_{0}\right)-A\left({\hat{\theta }}_{\lambda }-\theta_{0}\right)\right\}+\sqrt{N}\partial L\left(\theta_{0}\right)+\sqrt{N}A\left({\hat{\theta }}_{\lambda }-\theta_{0}\right),$$

where $\sqrt{N}\partial L\left(\theta_{0}\right)$ is bounded in probability, $\sqrt{N}A\left({\hat{\theta }}_{\lambda }-\theta_{0}\right)\overset{D}{\to }\sqrt{N}W$ which is bounded in probability as well. By Theorem 1 of the work in [42],

$$\underset{\left|{\hat{\theta }}_{\lambda }-\theta_{0}\right|\leq M/\sqrt{N}}{\sup}\left|\partial L\left({\hat{\theta }}_{\lambda }\right)-\partial L\left(\theta_{0}\right)-A\left({\hat{\theta }}_{\lambda }-\theta_{0}\right)\right|=o_{p}\left(\frac{1}{\sqrt{N}}\right).$$

Therefore, $\sqrt{N}\left\{\partial L\left({\hat{\theta }}_{\lambda }\right)-\partial L\left(\theta_{0}\right)-A\left({\hat{\theta }}_{\lambda }-\theta_{0}\right)\right\}\overset{p}{\to }0$. Finally, $\sqrt{N}\left|\partial_{j}L\left({\hat{\theta }}_{\lambda }\right)\right|$ is bounded and the right side of (A14) converges to 0, which proves $\mathrm{pr}(j\in T_{N})\to 0$ for j∉T.  □

## References

[1] Little R.J., Rubin D.B. (2002). Statistical Analysis with Missing Data.

[2] Shao J., Zhao J. (2013). Estimation in longitudinal studies with nonignorable dropout. Stat. Its Interface.

[3] Wang S., Shao J., Kim J.K. (2014). An instrumental variable approach for identification and estimation with nonignorable nonresponse. Stat. Sin..

[4] Zhao J., Shao J. (2015). Semiparametric pseudo-likelihoods in generalized linear models with nonignorable missing data. J. Am. Stat. Assoc..

[5] Miao W., Tchetgen Tchetgen E.J. (2016). On varieties of doubly robust estimators under missingness not at random with a shadow variable. Biometrika.

[6] Zhao J., Ma Y. (2018). Optimal pseudolikelihood estimation in the analysis of multivariate missing data with nonignorable nonresponse. Biometrika.

[7] Miao W., Liu L., Tchetgen Tchetgen E., Geng Z. (2019). Identification, Doubly Robust Estimation, and Semiparametric Efficiency Theory of Nonignorable Missing Data With a Shadow Variable. arXiv.

[8] Tchetgen Tchetgen E.J., Wirth K.E. (2017). A general instrumental variable framework for regression analysis with outcome missing not at random. Biometrics.

[9] Sun B., Liu L., Miao W., Wirth K., Robins J., Tchetgen Tchetgen E.J. (2018). Semiparametric estimation with data missing not at random using an instrumental variable. Stat. Sin..

[10] Zhao J., Yang Y., Ning Y. (2018). Penalized pairwise pseudo likelihood for variable selection with nonignorable missing data. Stat. Sin..

[11] Jiang W., Bogdan M., Josse J., Miasojedow B., Rockova V., Group T. (2019). Adaptive Bayesian SLOPE–High-dimensional Model Selection with Missing Values. arXiv.

[12] Jiang W., Josse J., Lavielle M., Group T. (2020). Logistic regression with missing covariates—Parameter estimation, model selection and prediction within a joint-modeling framework. Comput. Stat. Data Anal..

[13] Johnson A.E., Pollard T.J., Shen L., Li-wei H.L., Feng M., Ghassemi M., Moody B., Szolovits P., Celi L.A., Mark R.G. (2016). MIMIC-III, a freely accessible critical care database. Sci. Data.

[14] Zhao J., Ma Y. (2019). A versatile estimation procedure without estimating the nonignorable missingness mechanism. arXiv.

[15] Liang K.Y., Qin J. (2000). Regression analysis under non-standard situations: A pairwise pseudolikelihood approach. J. R. Stat. Soc. Ser. B.

[16] Zhao J., Shao J. (2017). Approximate conditional likelihood for generalized linear models with general missing data mechanism. J. Syst. Sci. Complex..

[17] Zhao J. (2017). Reducing bias for maximum approximate conditional likelihood estimator with general missing data mechanism. J. Nonparametr. Stat..

[18] Yang Y., Zhao J., Wilding G., Kluczynski M., Bisson L. (2020). Stability enhanced variable selection for a semiparametric model with flexible missingness mechanism and its application to the ChAMP study. J. Appl. Stat..

[19] Zhao J., Chen C. (2019). Estimators based on unconventional likelihoods with nonignorable missing data and its application to a children’s mental health study. J. Nonparametric Stat..

[20] Tibshirani R. (1996). Regression shrinkage and selection via the lasso. J. R. Stat. Soc. Ser. B.

[21] Fan J., Li R. (2001). Variable selection via nonconcave penalized likelihood and its oracle properties. J. Am. Stat. Assoc..

[22] Zhang C.H. (2010). Nearly unbiased variable selection under minimax concave penalty. Ann. Stat..

[23] Zou H. (2006). The adaptive lasso and its oracle properties. J. Am. Stat. Assoc..

[24] Cai T., Tian L., Wei L. (2005). Semiparametric Box–Cox power transformation models for censored survival observations. Biometrika.

[25] Kosorok M.R. (2007). Introduction to Empirical Processes and Semiparametric Inference.

[26] Minnier J., Tian L., Cai T. (2011). A perturbation method for inference on regularized regression estimates. J. Am. Stat. Assoc..

[27] Hu Z., Melton G.B., Arsoniadis E.G., Wang Y., Kwaan M.R., Simon G.J. (2017). Strategies for handling missing clinical data for automated surgical site infection detection from the electronic health record. J. Biomed. Inform..

[28] Li J., Wang M., Steinbach M.S., Kumar V., Simon G.J. Don’t Do Imputation: Dealing with Informative Missing Values in EHR Data Analysis. Proceedings of the 2018 IEEE International Conference on Big Knowledge (ICBK).

[29] Phillips A., Shaper A.G., Whincup P. (1989). Association between serum albumin and mortality from cardiovascular disease, cancer, and other causes. Lancet.

[30] Katz S., Klotz I.M. (1953). Interactions of calcium with serum albumin. Arch. Biochem. Biophys..

[31] Butler S., Payne R., Gunn I., Burns J., Paterson C. (1984). Correlation between serum ionised calcium and serum albumin concentrations in two hospital populations. Br. Med. J..

[32] Hossain A., Mostafa G., Mannan K., Prosad Deb K., Hossain M. (2015). Correlation Between Serum Albumin Level and Ionized Calcium in Idiopathic Nephrotic Syndrome in Children. Urol. Nephrol. Open Access. J..

[33] Kroll M., Elin R. (1985). Relationships between magnesium and protein concentrations in serum. Clin. Chem..

[34] Huijgen H.J., Soesan M., Sanders R., Mairuhu W.M., Kesecioglu J., Sanders G.T. (2000). Magnesium levels in critically ill patients: What should we measure?. Am. J. Clin. Pathol..

[35] Djagbletey R., Phillips B., Boni F., Owoo C., Owusu-Darkwa E., deGraft Johnson P.K.G., Yawson A.E. (2016). Relationship between serum total magnesium and serum potassium in emergency surgical patients in a tertiary hospital in Ghana. Ghana Med. J..

[36] Luo X., Tsai W.Y. (2011). A proportional likelihood ratio model. Biometrika.

[37] Shao J. (2003). Mathematical Statistics.

[38] Arcones M.A. (1998). Weak convergence of convex stochastic processes. Stat. Probab. Lett..

[39] Rejchel W. (2017). Model selection consistency of U-statistics with convex loss and weighted lasso penalty. J. Nonparametric Stat..

[40] Geyer C.J. (1994). On the asymptotics of constrained *M*-estimation. Ann. Stat..

[41] Pflug G.C. (1995). Asymptotic stochastic programs. Math. Oper. Res..

[42] Niemiro W. (1993). Least empirical risk procedures in statistical inference. Appl. Math..

